# Death of a Surgeon from Prussic Acid

**Published:** 1845-04

**Authors:** 


*Death of a Surgeon from Prussic Acid.*—A lamentable occurrence took place at Stratton, near Cirencester, on Thursday, the 23rd ult., whereby Mr. Daniel Stuart Holmes, one of the surgeons to the union, came to an untimely end, from accidentally taking an overdose of prussic acid. An inquest was held before Mr. Ball, on Friday. The particulars are as follows :—Mr. Holmes left his house on horseback, on the afternoon of Wednesday, on professional business. At about four, he returned, went into the house, and complained of being much fatigued. After some cheerful conversation, he expressed a wish to be left alone, as he was sleepy. His brother, Mr. James Holmes, and deceased, had some conversation on their professional business, and he was then, at his own request, left to his repose till about six o'clock. His servant said he was then sitting in his chair, leaning back, and did not seem well. She said "You had better go to bed. As I left the room, I saw him go out of the opposite door to that at which I was leaving, into the surgery. I returned almost immediately, and as I came into the room, I heard a noise as if he were sick; at the same time I heard something drop to the ground. That was the bottle now produced. I looked towards deceased, and saw him falling. He put his arms out to save himself. He fell rather forward. I ran to deceased, and lifted him up, and fetched a jug of water, of which I poured some down his throat, and threw some over his face. I then told the boy to run to Mr. James Holmes, and fetch him, as he had gone out. He came home immediately. This was ten minutes from the time of deceased falling. He gave him an emetic. Deceased's pulse was then hardly perceptible. As soon as the boy had returned from calling Mr. James Holmes, I sent him to Cirencester for Dr. Kenneir, but deceased was dead before he could get there." He breathed his last about twenty minutes or half an hour after his housekeeper heard him fall to the ground. The bottle was labelled, "Hydrocyanic acid, of Scheele's strength. Minor dose, one drop." The bottle was a little less than half full when picked up. It would have held half an ounce more. The cork was in. Mr. Holmes had killed a cat with a little of it a short time before. She did not know if he had been in the habit of taking any of it himself. Mr. James Holmes said his brother occasionally complained of a pain in his head, and occasionally in his right side. He had also a slight cough. He took medicine every week. Frequently, when he had complained of pain, he had seen him take a little prussic acid by touching his tongue with the cork of that bottle. About six months ago deceased had been heard jocularly to remark upon the strength of the dose he had taken, as it had made him giddy. He was as cheerful as he had ever been in his life on the evening of his death. Dr. Kenneir and Mr. Pooley were requested to make a post-mortem examination of the body. They had no doubt deceased died from taking prussic acid. The remedies administered were brandy and ammonia. The jury, after a short consultation, returned a verdict to the effect, "that deceased had died from accidentally taking an overdose of prussic acid."—*Wilts and Gloucestershire Standard.*

